# Neural Tube Organoids: A Novel System to Study Developmental Timing

**DOI:** 10.1007/s12015-024-10785-5

**Published:** 2024-09-04

**Authors:** Alexa Rabeling, Amy van der Hoven, Nathalie Andersen, Mubeen Goolam

**Affiliations:** 1 Department of Human Biology, Faculty of Health Sciences, University of Cape Town, Cape Town, 7925 South Africa; 2 UCT Neuroscience Institute, Cape Town, South Africa

**Keywords:** Neural tube development, Allochrony, Neural tube organoids, Development modelling, Stem cells

## Abstract

The neural tube (NT) is a transient structure formed during embryogenesis which develops into the brain and spinal cord. While mouse models have been commonly used in place of human embryos to study NT development, species-specific differences limit their applicability. One major difference is developmental timing, with NT formation from the neural plate in 16 days in humans compared to 4 days in mice, as well as differences in the time taken to form neuronal subtypes and complete neurogenesis. Neural tube organoids (NTOs) represent a new way to study NT development *in vitro.* While mouse and human NTOs have been shown to recapitulate the major developmental events of NT formation; it is unknown whether species-specific developmental timing, also termed allochrony, is also recapitulated. This review summarises current research using both mouse and human NTOs and compares developmental timing events in order to assess if allochrony is maintained in organoids.

**Graphical Abstract:**

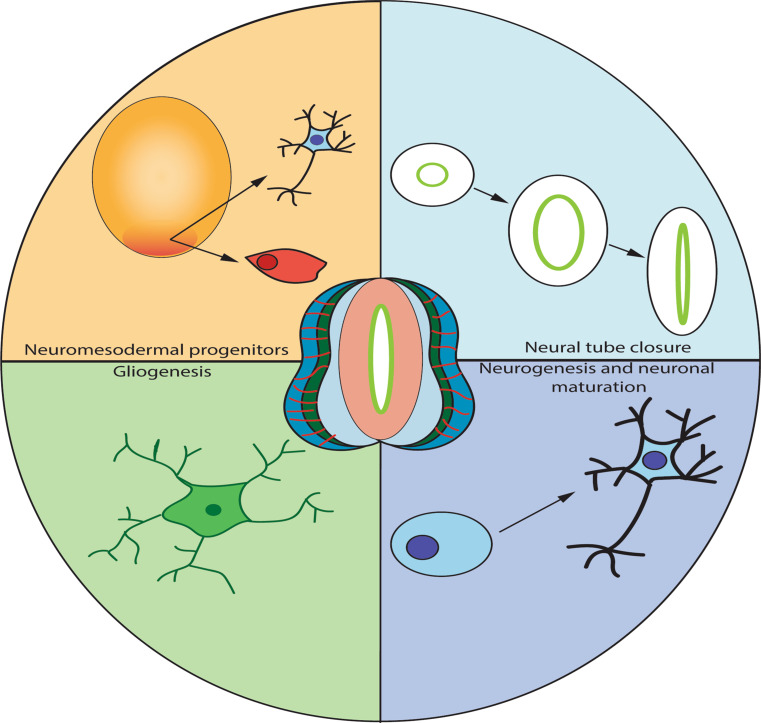

## Introduction

While the molecular mechanisms and underlying developmental processes that occur during embryogenesis are largely conserved between species the rate and timescale of these processes is species-specific and can differ dramatically. This is especially true when comparing timing mechanisms during central nervous system (CNS) development in rodents and humans which might underlie the differences in relative morphogenetic complexity and be key to understanding neurogenesis [1–3].

These timing discrepancies, termed allochrony, are not attributed to varying sensitivities to cell signals nor to differing gene expression or gene regulatory elements, but rather from poorly understood cell-autonomous mechanisms [4, 5] and is particularly evident when comparing rodent and human neuronal development. The most easily recognisable form of this discrepancy is present in the segmentation clock, in which oscillatory gene expression regulates the sequential appearance of paired somites in the developing embryo [6]. The oscillation period describes the duration of one oscillation cycle and is species-specific, thus serving as a significant point of interest in the comparison between developmental timing in mice and humans [6, 7]. The oscillation period is approximately 2–3 h in mice and 5–6 h in humans [4, 8–11]. Neurulation, the transformation of the neural plate into the neural tube, is another such process that differs in timing between rodents and humans. In mice the entire process takes 4 days to complete while in humans this same developmental task takes 16 days [12, 13], 4 times longer than in mice. Similarly, the formation of neural progenitors takes about 3 times longer in humans than in mice [5, 14, 15] while the differentiation of motor neurons (MNs), a subtype of post-mitotic neurons in the spinal cord neuronal subtype found in the spinal cord, takes up to 4 days in mice but up to 2 weeks in humans [5, 16, 17], likely due to differences in protein turnover between species [5].

While comparative studies in rodent and human embryos have been an excellent model to study the differences in neuronal developmental timing, a detailed molecular characterisation of in vivo events is at best extremely challenging both experimentally and ethically. Additionally, species-specific variances in developmental timing pose a significant challenge when attempting to use these systems to understand human biology. There is thus a need to develop more accurate alternative models of human neurulation. The recent emergence of a novel in vitro 3D model of neuronal development, so called neural tube organoids (NTOs), which are able to model the embryonic precursor stages of the CNS, has the potential to circumvent these concerns and be a critical resource in understanding both developmental allochrony as well be the de facto model in which to study human neurogenesis.

In this review, we outline the discoveries made using NTOs in elucidating the temporal differences between rodent and human neuronal development. We compare the results found in both mouse and human neural tube organoids as well as the relative timing of the emergence of neuromesodermal progenitors (NMPs), neural tube closure (NTC), neuronal maturation, and glial cell formation in this model system.

### Formation of Neural Tube Organoids

**Fig. 1:**
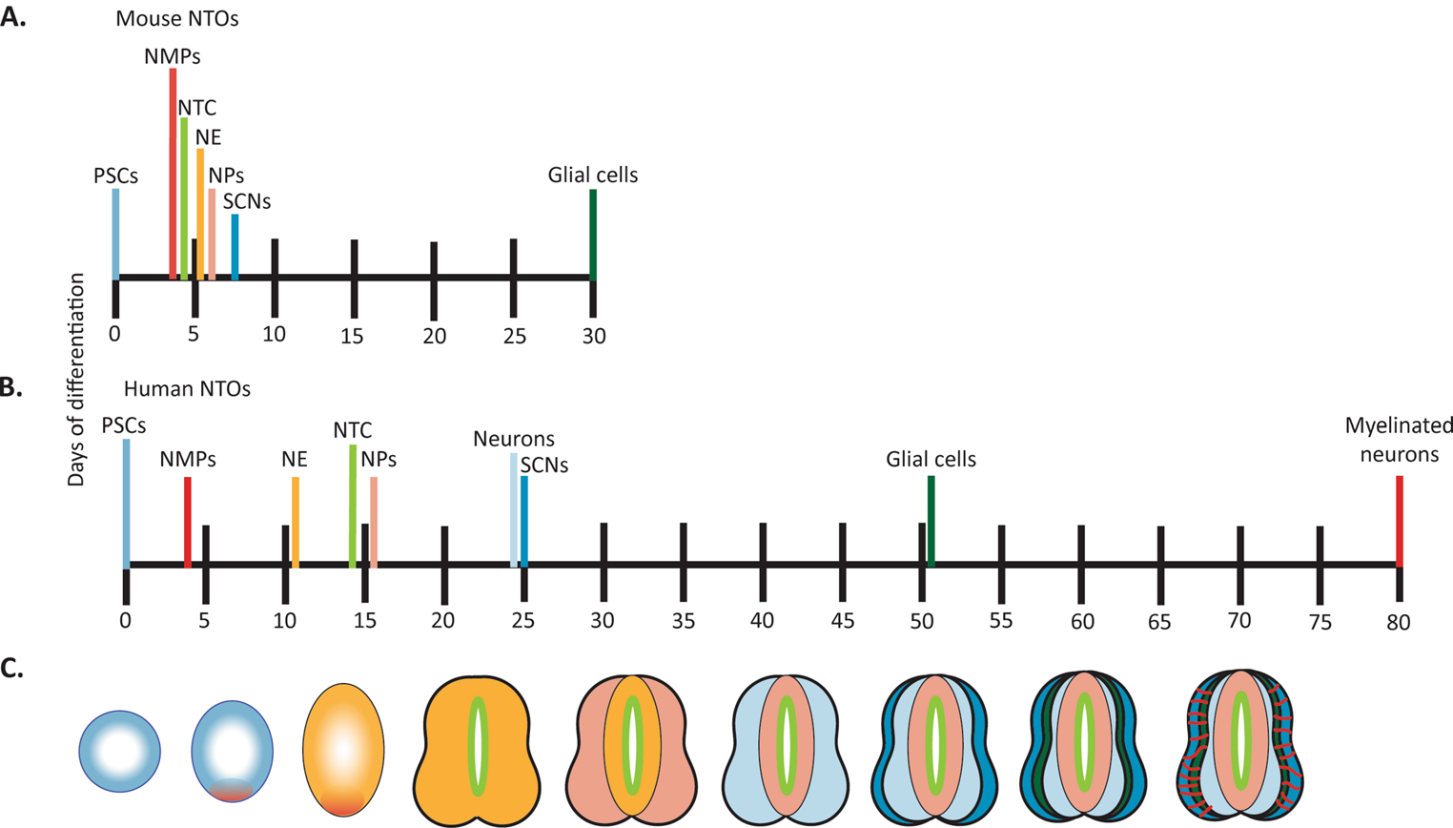
Summary of the timing of major developmental events in mouse and human NTOs. (**A**) Timing of emergence of neural tube cell types and other major developmental events taking place in mouse NTOs, with timeline indicating days of differentiation taken for each cell type to form/event to occur. (**B**) Timing of emergence of neural tube cell types and other major developmental events taking place in human NTOs, with timeline indicating days of differentiation taken for each cell type to form/event to occur. In both A and B, timepoint represents the average time taken in all papers discussed for each species. (**C**) Schematic representing development of NTOs, with major cell types and developmental events colored according to the bars in A and B. PSCs = pluripotent stem cells; NMPs = neuromesodermal progenitors; NTC = neural tube closure; NE = neuroectoderm; NPs = neural progenitors; SCNs = spinal cord neurons

NTOs are 3D in vitro cell aggregates which self-organize and proliferate to recapitulate in vivo neural tube development [18]. These organoids are cultured from pluripotent stem cells, such as induced pluripotent stem cells (iPSCs), embryonic stem cells (ESCs), or adult stem cells (ASCs), which are then induced to aggregate and to undergo differentiation into neural cell fate lineages. This process is facilitated by extracellular matrices (ECMs) such as Matrigel, and supplementation with required signaling factors to drive differentiation into CNS cell types [19]. These organoids recapitulate major developmental stages of neural tube development in vitro [18–22] and, with the advent of human induced pluripotent stem cells (hiPSCs) can be utilized to form human NTOs to model human neural tube (NT) development [22, 23], overcoming the ethical constraints of studying these events in the embryo proper (Fig. 1).

The first NTOs were formed using mouse embryonic stem cells (mESCs) [19–21, 24, 25]. In 2014, Meinhardt et al. formed neuroepithelial cysts by embedding single mESCs in Matrigel, and found that neuroectoderm (NE) identity, which emerges at embryonic day (E) E7.5 in the mouse embryo [23], was seen from 96 h of differentiation as seen by *Sox1* expression, which increased until 144 h [19] (Table 1). The authors determined that neural differentiation, in this case, followed a primitive ectoderm-like stage in which the expression of pluripotency markers *Oct4* and *Nanog* decreased in the first 96 h of differentiation concomitantly with *Fgf5* being expressed from 72 until 120 h, which was then followed by *Sox1* expression [19], the first pan-neuroectodermal marker in mice [23]. Following a similar protocol in which single mESCs were embedded in Polyethylene glycol (PEG)-based hydrogels, with individual components of Matrigel conjugated in order to test their relative contributions to neural morphogenesis, Ranga et al. (2016) found that Sox1 was strongly expressed from day 5 as indicated by green fluorescent protein (GFP) expression from a Sox1-GFP transgene in their neuroepithelial cysts [24]. When modifying their hydrogels, Ranga et al. (2016) found that a PEG-based hydrogel of intermediate stiffness (defined here as 4 kPa), generated with matrices insensitive to degradation by metalloproteases (MMPs) by adding laminin, resulted in the most robust Sox1 expression on day 5 of culture [24]. NTOs were formed in a similar manner by Park et al. (2022), with single mESCs being embedded in Matrigel, and were first induced to differentiate into an epiblast-like state, followed by neural induction, with expression of neuroectodermal genes *Sox1* and *Pax6* being significantly expressed from 144 h [18], equivalent to E8.0 in vivo [26]. However, in both cases, no evidence of NMPs (which emerge at E7.5 in vivo [26]) was seen. In the case of Meinhardt et al. (2014), this was due to the neuroepithelial cysts having an anterior identity rather than modelling the posterior end of the neural tube [19]; however, Park et al. (2022) saw anterior-posterior patterning in their elongating NTOs, with some cells acquiring spinal cord identity as determined by Hox gene expression [18]. This is in contrast to research by Veenvliet et al. (2020), in which cells with neural tube identity arose at least partially from a NMP population which was detected at 96 h via immunostaining for the co-expression of Sox2 and Brachyury to show the NMP population and 120 h via single cell RNA sequencing (scRNA-seq) [20, 22]. The lack of NMPs in NTOs generated by Park et al. (2022) is interesting to note, as it is thought that cells of the posterior neural tube, especially those that go on to form the spinal cord, arise from NMPs and additionally drive extension of the tailbud in vivo [27–29]. Taking this into account, the formation of elongating NTOs with cells of a posterior spinal cord identity that are seen to differentiate from epiblast-like cells generally, and not NMPs specifically may potentially indicate another differentiation pathway to form posterior NT cells, or a deviation of NTOs from in vivo growth patterns. Interestingly, both Park et al. (2022) and Meinhardt et al. (2014) formed NTOs by embedding single mESCs in extracellular matrix [18, 19], while Veenvliet et al. (2020) formed their organoids by forming mESC aggregates, utilizing 200–250 cells per aggregate, and observed cells of NE identity from 96 h (NE cells arise on E7 in vivo [26]) [20]. The distinction of whether single cells or aggregates were used to form NTOs is important, as starting cell numbers can influence differentiation dynamics [30, 31]. Additionally, Veenvliet et al. (2020) used Chiron in their differentiation procedure [20], which is a well-known posteriorizing marker [32–34] and has been shown to contribute to NMP formation in vitro [32, 34].

**Table 1:** Overview of timing taken to reach neural tube developmental timepoints in mouse and human neural tube organoids

Paper	Species (Mouse/Human); if human (hESCs/hiPSCs)	Total length of culture period (days)	Days to NMP emergence	Days to NE emergence	Days to NP emergence	Days to mature neuron formation (by TUBB3 expression)	Days to formation of SCNs	Days to formation of glial cell types	Lumen formation (Y/*N*) and number of days to lumen formation	Type of lumen formed (single/multiple and apico-basal polarity/none)
Meinhardt et al. [19]	Mouse	9	None	5	6	7	7	None	Yes; 5	Single; apico-basal polarity
Demers et al. [25]	Mouse	9	N/A	N/A	6	N/A	6	None	None	N/A
Ranga et al. [24]	Mouse	9	N/A	5	7	5	7	None	Yes; 5	Single; apico-basal polarity
Veenvliet et al. [20]	Mouse	5	4	5	5	N/A	None	None	Yes; 5	Single; apico-basal polarity
Park et al. [18]	Mouse	10 NCCs cultured until day 30	None	6	6	10	7	30 (after further culturing of NCCs generated by NTOs)	Yes; 5	Single; apico-basal polarity
Duval et al. [21]	Mouse	7	N/A	5	4	N/A	7	N/A	None	N/A
	Human (hiPSCs)	14	N/A	7	9	N/A	14	N/A	None	N/A
Ogura et al. [36]	Human (hESCs and hiPSCs)	48 Dissociation culture until day 112	None	6	15	29	24	39	Yes; 15	Continuous epithelial structure; improper apico-basal polarity
Hor et al. [41]	Human (hiPSCs)	42	N/A	10	10	14	21	42	Yes (rosettes); 21	Rosettes; apico-basal polarity
Zheng et al. [45]	Human (hESCs)	25	N/A	8	9	9	18	N/A	Yes; 8	Single; apico-basal polarity
Fedorova et al. [48]	Human (hESCs and hiPSCs)	12	N/A	10	12	N/A	N/A	N/A	Yes (rosettes); 10	Multiple; apico-basal polarity
Martins et al. [38]	Human (hESCs and hiPSCs)	150	3	5	10	50	20	50; significant proportion at day 150	None	N/A
Karzbrun et al. [42]	Human (hESCs and hiPSCs)	9	N/A	4	N/A	N/A	N/A	N/A	Yes; 5	Single; apico-basal polarity
Libby et al. [37]	Human (hiPSCs)	10	6	7	10	10	N/A	N/A	Yes; 7	Multiple; apico-basal polarity
Lee et al. [35]	Human (hESCs)	171	2	7	30	30	30	66	Yes; 7–8	Multiple; apico-basal polarity
Zou et al. [40]	Human (hiPSCs)	63	N/A	14	11	N/A	25	N/A	None	N/A
Xue et al. [39]	Human (hESCs and hiPSCs)	56	N/A	14	21	21	42	21	None	N/A
Chooi et al. [44]	Human (hiPSCs)	120	N/A	30	30	30	30	90	Yes; 30	Not checked
Balashova et al. [43]	Human (hiPSCs)	21	N/A	18–21	N/A	N/A	N/A	N/A	Yes; 18–21	Multiple; apico-basal polarity

hESCs = human embryonic stem cells; hiPSCs = human induced pluripotent stem cells; NMP = neuromesodermal progenitor; NE = neuroectoderm; SCNs = spinal cord neurons

### Emergence of Human Neural Tube Organoids

NTOs formed using human stem cells (human embryonic stem cells (hESCs) or human induced pluripotent stem cells (hiPSCs) have exclusively been derived from aggregates of pluripotent stem cells [21, 22, 35–45], rather than by embedding single cells into ECM substitutes. The number of cells used varies widely between protocols, ranging from 3 × 10^3^ cells per aggregate [37] up to 1 × 10^5^ cells per aggregate [39] with the median number of cells used being 9 × 10^3^ cells per aggregate [36, 38, 40, 43]. Uniquely, some human NTO (hNTO) protocols make use of a 2D to 3D approach, in which colonies plated onto ECM replacements such as Matrigel or Geltrex are lifted into suspension to generate 3D structures [35, 42], rather than dissociating then re-aggregating cells to form embryoid bodies (EBs). This approach was also used by Tidball et al. (2023) in order to create human cortical organoids which only contained one rosette structure by day 5, thereby better recapitulating the folding of the NE into the NT as it relates to brain development [46]. NE formation was observed in all hNTOs, with induction of NE genes seen after 5 days of differentiation by Martins et al. (2020) as indicated by protein expression of SOX1 and mRNA expression of *SOX2* and *PAX6*, as determined by scRNA-seq data [38]; as well as by Karzbrun et al. (2021), who saw high levels of protein and mRNA expression of *PAX6* and *SOX2* after 5 days of differentiation utilising immunostaining and scRNA-seq [42]. Cells of NE identity (normally arising four weeks post conception at Carnegie stage (CS) 9 in vivo [47]) were only seen later in most cases, with some identifying NE cells after 6 [22, 36], 7 [21, 35], 8 [45] or 10 [37, 48] days of differentiation. In a few cases, NE cells were only observed at much later stages, with Zou et al. (2022) observing *SOX2* mRNA expression from 14 days [40], Balashova et al. (2024) identifying SOX2 positive cells by immunostaining at 18 days [43], Xue et al. (2023) observing PAX6 positive cells on day 21 by immunostaining [39] and Chooi et al. (2023) observing high levels of SOX1 expression around neural rosettes by immunostaining at day 30 [44]. These observed differences in the emergence of neuroectodermal fate could be due to various factors, one of them being that the timepoints at which organoids are analysed are chosen for a variety of reasons, and unless neuroectodermal formation is being specifically assessed, the start of this process may not be looked for by the authors. For this reason, and considering that PAX6 is the earliest NE marker in humans [49], we chose to exclude some papers when determining an overall timing for NE induction in hNTOs [21, 41, 43, 44]. After exclusion of these papers, we found the average time of NE formation to be 10 days, the median time to be 8 days, and the minimum and maximum number of days being 4 and 30, respectively.

Most research groups have focused on either using mouse or human pluripotent cells in their organoid protocols making comparisons between developmental timing difficult. However, Duval et al. (2020) directly compared the developmental timing in mouse and human spinal organoids (mSOs/hSOs) and found that dorsal neural tube markers were present from day 4–5 in mSOs, while hSOs only showed positive expression between day 7–9 [50]. Differentiation into dorsal interneurons took approximately 7 days in mSOs and 14 days in hSOs [50]. Overall, the authors concluded that, while both mSOs and hSOs showed similar developmental trajectories differentiation events took approximately 2.5x longer in SOs generated from human cells than those generated from mouse cells [50].

### Neuromesodermal Progenitor Formation in Human and Mouse Neural Tube Organoids

NMP formation in hNTOs is generally seen when protocols utilize posteriorizing agents such as Chiron, thereby inducing spinal cord fates. During in vivo development, the primitive streak gives rise to the bipotent NMP cell population which are able to develop into both neural and mesodermal cell types and contribute to both the spinal cord and paraxial mesoderm [28]. Lee et al. (2022) treated their hESCs with Chiron during the 2D induction stage, along with SB-432,542, a small molecule antagonist of the TGF-beta signalling pathway [51], which together led to the emergence of NMPs (which in vivo arises between 26 and 30 days at CS12 [52]) after 2 days of differentiation [35]. Similarly, Martins et al. (2020) treated their organoids with Chiron, but in this case paired it with bFGF and saw the emergence of an NMP population on day 3 of culture (typically observed at E7.5 in vivo [53]) [38]. In both cases, the presence of NMPs was determined by co-staining for SOX2 and BRACHYURY, the co-expression of which is a defining feature of NMPs [20].

### Neural Tube Closure in Human and Mouse Neural Tube Organoids

The occurrence of an NTC-like process in NTOs is an essential aspect of their application in the study of neural tube defects (NTDs). During neurulation in the human embryo, the ends of the neural folds do not initially fuse, which leaves two openings, known as neuropores, in the cranial and caudal ends of the tube called the rostral and caudal neuropore, respectively [54]. Over time, these neuropores will reduce in size and fuse completely during neural tube closure, with the rostral neuropore closing before the caudal neuropore during the fourth week of development. In contrast, mice undergo neural tube closure at multiple points, giving rise to several neuropores. This process is initiated on embryonic day 8.5, starting with the closure at the hindbrain and cervical boundary. The second closure occurs at the forebrain and midbrain boundary, followed by the third closure at the rostral end of the forebrain. As a result, three neuropores are formed within the mouse embryo - the hindbrain and anterior neuropores in the cranial region and the posterior neuropore in the lower spinal region. The closure of the neuropores is facilitated by the surface ectoderm and marks the end of primary neurulation. Later on in development, the rostral and caudal ends of the neural tube will form the brain and the spinal cord, respectively [54].

In mouse NTOs (mNTOs), a lumen or cavity is formed which resembles the in vivo lumen of the developing NT, a single lumen with apico-basal polarity. In embryos, lumen formation typically commences between E8.5 and E9.5 in vivo, demonstrating apico-basal polarity between E8.5 and E10.5 [26]. Generally, in mNTOs, fully formed lumen were observed after 120 h of differentiation [18–20, 24], with lumen beginning to form at 96 h in one protocol and only gaining apico-basal polarity at 144 h, although lumen formation in this case was likely due to the acquisition of epithelial fate from an earlier ectodermal developmental stage [19], rather than a true NTC-like process. However, from the data, it cannot be concluded whether lumen formation occurred due to primary neurulation (i.e. NTC) or secondary neurulation. The formation of luminal structures is much more varied in hNTOs, with some forming no lumen [21, 38–40], others forming multiple apico-basally oriented lumen [37], or rosette-like structures [55], and yet others forming epithelial structures with the apical side being found on the outside of the structure, rather than internally as would be expected [36]. In hNTOs in which single lumen did form with the expected apico-basal polarity, this occurred by day 5 [22] day 8 [45] of differentiation, or even as late as 21 days [43] (these processes occur between days 25–28 in vivo [56]). Lee et al. (2022) and Karzbrun et al. (2021) looked closely at the formation of lumen in their NTOs to assess the NTC-like process which occurred [35, 42], modelling primary neurulation. Lee et al. (2022) utilized ZO-1 staining to stain for apical surfaces and identified folding of neuroectodermal cells from day 11, with fully closed lumen observed on day 15 [35]. Karzbrun et al. (2021) generated a sophisticated culture system in which hiPSCs were seeded onto micropatterns, generating a 2D micropatterned culture which was driven to become 3D after the addition of Matrigel on day 2, a process which took 2 days and resulted in 3D pluripotent stem cell tissue with a single lumen [42]. From day 5, this 3D tissue was exposed to the morphogens BMP4 and SB431542. On day 7, i.e. 2 days after morphogen addition, this pluripotent tissue had differentiated into neuroectodermal tissue covered by surface ectoderm, which started bending, with closure being completed 4 days after morphogen addition [42]. From start to finish, this process took approximately 5 days [42], about half the time that NTC takes in vivo [7]. This folding process was initiated at two points, resembling hinge points in the in vivo neural tube, with fusion being initiated by surface ectoderm and an actin-enriched zipper appearing at fusion sites from days 3 to 4 [42].

On the other hand, Fedorova et al. (2019) saw formation of lumen in their neural rosettes between 8 and 10 days and concluded that this occurred via a secondary neurulation-like process, as the mesenchymal-epithelial transition markers SNAIL and VIMENTIN, were expressed on day 2, followed by a decrease on day 3 as PSA-NCAM moved from a lower to a higher molecular weight [48], which are key aspects of secondary neurulation in vivo and occurs between gestational weeks 5 and 6 [57–60]. Neural rosette formation was also seen at 21 days by Hor et al. (2018) [41] and at 30 days in NTOs generated by Chooi et al. (2023) [44], with no indication of what was driving this process beyond neural differentiation. Rosette formation recapitulates some aspects of NT formation and can be thought of as a 2D cross-section of the 3D NT and thus can act as a proxy of NTC in vitro, or alternatively may more closely resemble secondary neurulation [48, 61].

While it is clear that the protocol by Karzbrun et al. (2021) represents the best model of human NTC, and was able to recapitulate NTDs [42], it is important to note that this is still not a full model of the developing NT, as no neurons were generated. It does, however, show that many factors, such as initial size, mechanical forces and interaction with surface ectoderm all play essential roles in the recapitulation of NTC in vitro.

### Neuronal Maturation in Human and Mouse Neural Tube Organoids

Later neural differentiation, for instance into post-mitotic neurons, has not been fully assessed in mNTOs. This is due to the relatively short culture period of most of these NTOs, although some neuronal differentiation still occurs as only 9–10 days are required for full neural maturation in mouse embryos [62]. Meinhardt et al. (2014) cultured their NTOs for 9 days [19]. Within this 9-day culture period, some neuronal differentiation was observed, with immunostaining revealing the presence of Neun, Tubb3 and Map2a positive neurons on days 7 and 8, Olig2 positive MN progenitors (pMNs) on days 6 and 7 and Isl1/2 positive MNs, which normally arise between E9.5 and E10.5 in vivo [63, 64] also on day 7 [19]. Finally, on day 9, six layers of pseudostratified progenitors and four neuronal cell layers were observed [19]. Similarly, utilizing their optimized hydrogel, Ranga et al. (2016) saw expression of Olig2, Nkx6.1 and Isl1 on day 7 [24]. They also saw expression of Tubb3 as early as 5 days of differentiation, at which point positive staining was seen on the periphery of the cysts, while on day 9, the cysts showed complete envelopment with Tubb3 staining as well as the presence of projections [24]. When Park et al. (2022) cultured their mNTOs until day 10, they used time-lapse imaging and found that mature neurons (present from E11 in vivo [65]) were present from day 8 [18]. Immunostaining for Brn3a determined that these neurons have a default dorsal state, with scRNA-seq on day 10 revealing that dorsal interneuron types 1, 2 and 3 (generated between E10.5 and E12.5 in vivo [65]) are present in these NTOs [18]. Since human neuronal maturation is a far longer process than that of mouse neuronal maturation, taking 110 days instead of 10 [62, 66], it is necessary to culture hNTOs for far longer periods of time to observe these cell types. The earliest emergence of differentiated neurons (arising at gestational week 5 in vivo [67]) in hNTOs as determined by Tubb3 expression was 9 days, although neurites were only observed on day 25 [45]. Similarly, Libby et al. (2021) saw Tubb3 positive neurons from day 10 of differentiation [37] and Hor et al. (2018) observed Tubb3 expression from day 14, which persisted at day 35 [41]. Both Lee et al. (2022) and Chooi et al. (2023) saw expression of Tubb3 at day 30 [35, 44] while Ogura et al. (2018) only observed this on day 40 [36]. While most papers assess neuronal differentiation by immunostaining for neuronal markers, Xue et al. (2023) analysed scRNA-seq data to determine the start of neuronal differentiation to be day 42, when *MAP2* and *TUBB3* were highly expressed [39]. Lastly, Martins et al. (2020) observed TUBB3 positive neurons on day 50 [38].

**Fig. 2:**
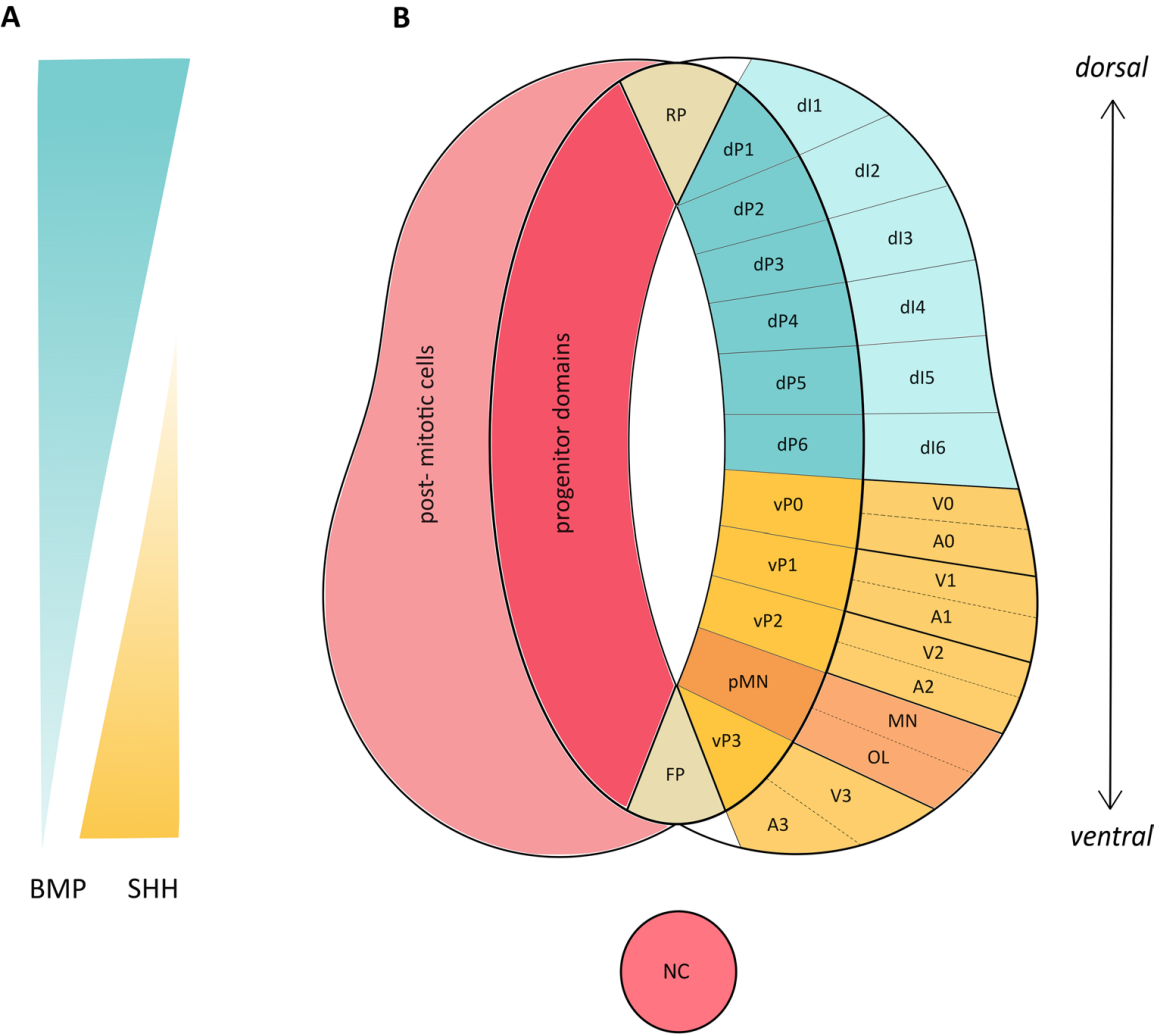
Dorso-ventral patterning of the developing mammalian neural tube, determined by SHH and BMPs, induces progenitor and post-mitotic neuronal fates. **(A)** SHH and BMP signalling gradients induce dorso-ventral patterning in the developing neural tube. SHH signalling is highest at the ventral floorplate, emanating from the notochord (NC), and decreases in intensity toward the dorsal roof plate. Ventral fates are induced by BMP signalling which is highest at the dorsal roof plate and decreases in intensity toward the floorplate. **(B)** Dorsal progenitor neurons (dP1-dP6) differentiate into dorsal interneuron populations (dI1-dI6), while ventral progenitors (vP0-vP3 and pMN) differentiate into ventral interneurons (V0-V3) and motor neurons (MN). These ventral progenitors later switch to the production of glial post-mitotic cells including astrocyte (A0-A3) and oligodendrocyte (OL) populations

In the developing spinal cord, the main neuronal subtypes that are formed can be divided into dorsal, intermediate, and ventral neurons, each with their own functions and gene expression patterns. In the dorsal spinal cord, most of the neurons can be described as sensory neurons, while the ventral spinal cord contains MNs together with ventral interneurons associated with motor function, and the interneurons in the intermediate spinal cord function to receive inputs from other areas of the spinal cord and to transmit them further [68, 69]. Neurons of all three regions are generated from dorsal and ventral progenitor domains, with intermediate interneurons being generated from a subset of both progenitor domain types and MNs being generated from their own progenitor layer [50]. Ogura et al. (2018) found that their hNTOs formed dorsal progenitors on day 15 of differentiation, with mature dorsal interneurons (present by gestational weeks 6–7 in vivo [70]), seen from day 24 expressing LBX1, indicating dI4-6 identity, and dorsal interneurons co-expressing BRN3 and ISLET1, indicating dI3 identity [36]. Upon BMP4 treatment, the complement of dI1 and dI2 neurons increased, as indicated by co-expression of BRN3A and LHX9 or BRN3 and LHX1, respectively [36], an expected result giving the importance of BMP signalling in specifying neural identity (Fig. 2). Zheng et al. (2019) found that their neural cysts had a default dorsal identity, with immunostaining revealing expression of progenitor markers PAX3, PAX6 and MSX1 from day 18 [45], which were also seen from scRNA-seq data to be expressed in hNTOs from 3 days in the case of *PAX3* and 5 days in the case of *MSX1* and *PAX6* [22], supporting the idea that the NT in vertebrates has a default dorsal neural identity [19, 45]. OLIG3 is another marker of dorsal progenitors, which was seen to be expressed after 21 days of differentiation by Xue et al. (2023); predictably, this was only seen in the outer layer of these organoids, as this region was not exposed to the SAG-releasing organizing centre, which activates Shh signalling [39] critical for neuronal identity along the dorso-ventral axis (Fig. 2). In terms of gene expression, scRNA-seq analysis identified neuronal cells containing the full complement of dorsal interneuron regional identities on day 42 [39]. Ogura et al. (2018) additionally saw expression of intermediate progenitor markers *PAX6*, *DBX1* and *DBX2* when treating their NTOs with 50nM of SAG as determined by RT-qPCR on day 15 [36].

Ventral neuron subtypes were found to be formed from ventral progenitors in a number of hNTOs, with Martins et al. (2020) observing expression of the ventral progenitor marker OLIG2 by immunostaining at day 10. Ventral interneurons co-expressing PAX2 and LHX1 were seen on day 50 [38] and CH10 positive V2a excitatory premotor interneurons neurons were generated on days 42 [41] and 50 [38] in different protocols. PCR analysis by Zou et al. (2022) indicated that mRNA expression of the ventral progenitor markers *NKX6.1* and *NKX6.2* were both highly expressed after 17 days of differentiation, although *NKX6.1* expression continued to increase until day 28, while *NKX2.2* expression decreased [40]. The decrease in Nkx2.2 along with the increase in Olig2 indicates differentiation into MN lineages by day 28. The increase in Nkx6.1 indicates the continued presence of ventral progenitors. Both Chooi et al. (2023) and Lee et al. (2022) saw emergence of interneurons after 30 days of differentiation, with Chooi et al. (2023) observing V1 and V2a interneurons as determined by CALB and CHX10 expression using immunostaining [44] and Lee et al. (2022) determining the presence of both interneuron types by scRNA-seq, and additionally saw the expression of *EVX1*, indicating that neurons of V0 identity were present in their NTOs [35]. Immunostaining, on the other hand, revealed the presence of glutamatergic V2a interneurons based on co-expression of LHX3 and VSX2 [35]. Xue et al. (2023) only saw neural progenitors expressing the ventral progenitor markers NKX6.1 and OLIG2 at day 21, although they found mature interneurons of the V0, V1 and V2a layers in their NTOs from day 42, 8 days earlier than Martins et al. (2020) [39].

MNs in the spinal cord arise at CS11 in vivo [63] and play essential roles in controlling movement and are all formed from the pMN domain in the ventral neural tube under the control of Shh signaling [50, 71]. Progenitor MNs co-expressing OLIG2 and NKX6.1 were found to be present after 15 [36] and 18 [45] days in hNTOs treated with Retinoic acid (RA) and either smoothened receptor agonist (SAG) [36] or Shh [45]; while secretion of SAG from an organizing centre resulted in the formation of these pMNs after 21 days [39]. These protocols resulted in formation of mature MNs co-expressing ISLET1/2 or ISLET1 and HB9 after 29 [36] and 18 [45] days, respectively, although emergence of MNs at 18 days only occurred with supplementation of BDNF, GDNF, CTNF, IGF-1, cAMP and ascorbic acid [45], which have been shown to drive MN formation in vitro [72]. Similarly to Ogura et al. (2018), treatment with RA and another Shh agonist, Purmorphamine, resulted in the formation of ISL1 positive MNs at day 28 of differentiation [41]. Additionally, Zou et al. (2022) utilized the protocol from Ogura et al. (2018) and found that the pMN markers Olig2 and Nkx6.1 showed peak expression at day 28, while the MN markers ChAT and Isl1 showed peak expression on day 43 [40]. Lee et al. (2022), who also utilized Purmorphamine in their culture, found that their protocol resulted in the generation of MNs on day 30 of differentiation, as determined by immunostaining for the co-expression of CHAT and ISL1, as well as by scRNA-seq data [35]. On the other hand, Martins et al. (2020) found that their neuromuscular organoids contained ISLET1 positive MNs on day 20, without the need for the addition of Shh signaling modulators, although it must be noted that the authors in this case started with an NMP population for their organoids [38], rather than hPSCs, representing a later developmental timepoint.

### Glial Cell Formation in Neural Tube Organoids

Beyond neurons themselves, supporting cells of the CNS known as glial cells are formed in the developing neural tube and spinal cord, the principal types of which are astrocytes and oligodendrocytes. Astrocytes have varied roles in the CNS, from neurometabolic coupling [73] to removal of excess neurotransmitters [74], and as such are essential to the health and activity of neurons themselves. Only one mNTO paper showed development of glial cells (which occur from E11.5 in vivo [75]), in which neural crest cells generated during the first 7 days of 3D differentiation were cultured for a further 30 days and were able to develop into Gfap and S100β expressing glial cells [18]. In hNTOs, astrocytes form readily, being observed in neuromuscular organoids from day 50, with GFAP positive astrocytes increasing from 14.2 to 58.2% at day 150 [38]. GFAP positive astrocytes were additionally observed from day 61 [35] and day 90 [44] in spinal cord organoids. Hor et al. (2018), on the other hand, utilized S100ß as a marker of astrocyte formation, concluding that astrocytes are present from day 35 of their culture [41]. While S100ß has been used as a marker of astrocytes for decades [76, 77], its presence in differentiating and mature oligodendrocytes [78, 79] mean that, on its own, it may not be a specific marker for astrocytes and so, in this case, it can only be concluded that glial cells were present in these NTOs. Oligodendrocytes myelinate the axons of neurons in the CNS by producing myelin basic protein (MBP) [80]. Martins et al. (2020) saw the earliest evidence of active oligodendrocytes (which emerge from roughly gestational week 17, in vivo [81]), with positive immunostaining for MBP being observed from day 50 [38]. In their spinal cord organoids, Lee et al. (2022) found that mature oligodendrocytes, as determined by CNPase immunostaining, were present from day 61, with myelination occurring at day 100 [35]. scRNA-seq data revealed that oligodendrocyte markers peaked at day 90 in spinal cord organoids generated by Chooi et al. (2023), with staining showing that MBP was being expressed to a small extent at the same stage, and increasing to abundance by day 120 [44].

## Conclusions and Future Perspectives

While still in its infancy, the results produced from research using NTOs from both mouse and human cell lines has been illuminating. From this body of work it can be deduced that NE formation begins at an average of 4 days in mNTOs and 8 days in human NTOs, indicating that this process takes twice as long in hNTOs compared to mNTOs, a similar finding to the 2.5-fold difference in developmental timing seen in other in vitro models [4, 5, 82]. In mNTOs, differentiated neurons were seen at an average of 7.5 days, while in hNTOs the average day of mature neuron differentiation was 27.7 (Fig. 1), meaning that it took 3.7x longer for neurons to emerge in hNTOs than in mNTOs, although it must be noted that most of these comparisons are not direct having been performed by different research groups, and do not take into account differences in protocols, cell lines and signaling molecules used, all of which could affect timing of differentiation.

However, in general, the data generated from neural organoids aligns with the generally accepted evidence indicating that in vivo neural tube developmental effects take on average 2.5x longer in humans than mice. The results generated from NTOs therefore generally align with in vivo developmental events in terms of timing indicating that, even in a fully in vitro environment, neural differentiation is a largely autonomous process that occurs on an intrinsic timeline dependant more on the species of the cells being used than on culture conditions. Culture conditions therefore instruct the differentiation pathway but are not a determinative factor in terms of the timing of differentiation events. The fact that cells can maintain a cellular ‘memory’ of developmental timing events through reprogramming and differentiation in the absence of any maternal factors underlines the accuracy of using stem cell models to study development. Critically, several groups have shown that both mouse and human NTOs recapitulate the major developmental events of neural tube formation, from neuroectoderm formation to neural progenitor differentiation and finally mature neuron formation. Furthermore, in hNTOs specifically, the emergence of different mature neuronal subtypes aligns with the timing of in vivo events. One aspect of neural tube formation which current human NTO protocols do not fully model is that of NTC. While the process of lumen formation was not fully characterized in many hNTOs, even when lumens with the correct polarity were formed, the sheer variance of lumen types that form in these organoids make it clear that future NTO protocols need to standardize lumen formation, with clear mechanisms on the kind of neurulation process, i.e. whether it is primary or secondary neurulation, that drives this in vitro.

Additionally, further standardization is required in NTO protocols themselves. Currently, NTO protocols are incredibly diverse in terms of what signaling factors are used, when they are applied, and what concentrations they are used at (Table 2). This is especially important to note because, while the effects of some signaling molecules seem to be the same across protocols, such as the use of Purmorphamine to generate MNs [35, 41, 44], the effects of other signaling molecules are less clear. To fully understand the effects of each signaling molecule on NT development in vitro, a more standardized approach to their generation is needed. Protocols also differ in terms of matrices, if and when they are used, which can greatly affect NTO development [20, 24, 44, 45]. Standardization when it comes to the use of matrices is important, as it is known that animal-derived matrices such as Matrigel can have high batch-batch variation thereby introducing variation into organoid cultures [83] and so the use of a standard, synthetic hydrogel could reduce not only batch-batch variation, but also improve replication between studies. These limitations provide important avenues for further research to make NTO models specifically, and organoid models generally, more robust and reliable.

**Table 2:** Overview of the use of supplements and matrices used in mouse and human neural tube organoid culture and the affected cell/tissue types

Paper	Species (Mouse/Human); if human (hESCs/hiPSCs)	Total length of culture period (days)	Cell/ tissue types formed	Culture supplements used (associated with cell types)	Timing (concentration)	General culture supplements used (concentraion)	Matrices used (concentration)
Meinhardt et al. [19]	Mouse	9	Neuroectoderm			N2 supplement; B27 supplement (both at 0.5X)	Matrigel (100%)
			General neural progenitors				
			pMNs and MNs (in larger patterned cysts)	Noggin and Retinoic acid	Day 0 for 48 h (75ng/ml) and Day 2 for 18 h (250nM)		
			Ventral (SHH and FoxA2 expressing) cells	SAG	Day 3 for 24 h (1µM)		
			Cervical (HoxC4 expressing) cells and ventral cells	Retinoic acid	Day 2 for 18 h (250nM)		
Demers et al. [25]	Mouse	9	MNs (upper spinal cord/ hindbrain)	Purmorphamine	Day 0 to 7 (3–4µM)	Retinoic acid (1µM)	Matrigel/ Geltrex in microdevice
Ranga et al. [24]	Mouse	9	Neuroectoderm	Retinoic acid	Day 2 to 3 (250nM)		PEG-based hydrogels
			pMNs and MNs				
Veenvliet et al. [20]	Mouse	5	Neuroectoderm			NDiff227 (supplemented with N2 and B27) Takara, Y40002	Matrigel (5%) from day 4
			NMPs	Chiron	Day 2 to 3 (3µM)		
Park et al. [18]	Mouse	10 NCCs cultured until day 30	Epiblast cells	bFGF and Activin A	Day 0 to 3 (12ng/ml and 20ng/ml)	N2 supplement (0.5X) and B27 supplement (1X)	Matrigel (100%)
			Neuroectoderm				
			Dorsal progenitors and interneurons				
			NCCs				
			Mature and peripheral neurons, glia, adipocytes and smooth muscle cells from further culture of NCCs				
Duval et al. [21]	Mouse	7	Neuroectoderm			N2 and B27 (without vitamin A) supplements (1X for both)	None
			Dorsal progenitors	FGF2 and Chiron	Day 0 to 3 (10ng/ml) and day 2 to 3 (3µM)		
			Dorsal interneurons	Retinoic acid and DAPT	Day 2 to 7 (10nM) and day 5 to 7 (10µM)		
			NCCs, roofplate cells, dorsal neurons and sensory neurons	BMP4	Various concentrations and timings; but generally day 3 to day 4 (5ng/ml)		
	Human (hiPSCs)	14	Neuroectoderm				
			Dorsal progenitors	Chiron	Day 0 to 4 (3µM)		
			Dorsal interneurons	Retinoic acid and DAPT	Day 2 to 9 (10nM) and day 9 to 14 (10µM		
			NCCs, roofplate cells, dorsal neurons and sensory neurons	BMP4	Various concentrations and timings		
Ogura et al. 36	Human (hESCs and hiPSCs)	48 Dissociation culture until day 112	Neuroectoderm	Y-27,632; Chiron; bFGF; SB431542 and retinoic acid	Day 0 to 3 (10µM); day 0 to 3 (3µM); day 0 to 3 (20ng/ml); day 0 to 6 (10µM) and day 3 to 15 (100nM)	N2 supplement (0.5X) and B27 supplement without vitamin A (1X) Ascorbic acid (0.5µM), BDNF (10ng/ml), 20 ng ml^− 1^ GDNF (20ng/ml) and retinoic acid (100nM) added for long-term culture	None
			Dorsal progenitors and dorsal interneurons (dI4-6)				
			Dorsal interneurons (dI1-3 and dI5)	BMP4	Day 15 to 24 (15ng/ml)		
			Ventral neurons	SAG (dose-dependent effect)	Day 3 to 15 (50nM or 500nM)		
Hor et al. [41]	Human (hiPSCs)	42	Neuroectoderm	LDN193189 and Chiron	Day 0 to 7 (10µM) and day 0 to 7 (unknown)	N2 supplement and B27 supplement (unknown) GDNF (10ng/ml); BDNF (10ng/ml) and Ascorbic acid (0.4 µg/ml) from day 17	Matrigel (100%) from day 10
			Thoracic MNs	Retinoic acid and Purmorphamine	Day 3 to 15 (1µM) and day 10 to 17 (1µM)		
			V2a interneurons	Purmorphamine	Day 10 to 17 (1µM)		
			Astrocytes				
Zheng et al. [45]	Human (hESCs)	25	Neuroectoderm	SB431542 and LDN193189	Day 1 to 18 (10µM) and day 1 to 18 (0.1µM	N2 supplement and B27 supplement (both at 0.5X)	Gel-3D condition: thick bed of Geltrex with 2% Geltrex supplemented into the medium from day 1
			Dorsal neurons	Default, but increased with Chiron treatment	Day 4 to 9 (3µM)		
			pMNs	Smoothened agonist and retinoic acid	Day 4 to 9 (1µM) for both		
			pMNs and further ventral subtypes	SAG; retinoic acid and SHH	Day 4 to 9 (1µM for SAG and retinoic acid) and day 4 to 9 (10nM)		
			ISLET1/2 and HB9 positive MNs	BDNF; GDNF; CNTF; IGF-1; cAMP and ascorbic acid	All from day 9 onwards (10ng/ml for BDNF, GDNF, CNTF and IGF-1); (1µM) and (0.2 µg/ml)		
			Cells of cervical identity	Retinoic acid	Day 4 to 9 (1µM)		
Fedorova et al. [48]	Human (hESCs and hiPSCs)	12	Neuroectoderm			N2 supplement and B27 supplement without vitamin A (both 0.5%) and Rock inhibitor (20µM); with Rock inhibitor removed from day 4	Cells plated in 2D on Matrigel
			NCCs				
Martins et al. 38	Human (hESCs and hiPSCs)	150	NMPs	Chiron and bFGF	Day 0 to 3 (3µM) and day 0 to 3 (40ng/ml)	N2 supplement and B27 supplement (1X for both)	None
			Neuroectoderm				
			Mesodermal pregenitors	bFGF; IGF1 and HGF	Day 0 to 2 (10ng/ml); day 0 to 4 (2ng/ml) and day 0 to 4 (2ng/ml)		
			Ventral progenitors				
			NCCs				
			pMNs and MNs				
			Myoblasts and contractile skeletal muscle cells				
			Glial cells				
Karzbrun et al. [42]	Human (hESCs and hiPSCs)	9	Neuroectoderm	SB431542	Day 2 to 9 (5µM)	N2 supplement (1%)	Cells plated onto pre-patterned dishes and 4% Matrigel added from day 4
			Surface ectoderm	BMP4	Day 5 to 9 (5ng/ml)		
Libby et al. [37]	Human (hiPSCs)	10	Neuroectoderm	SB431542 and LDN193189	Day 0 to day 7 (10µM) and day 0 to 7 (0.2µM)	N2 supplement; heparin (2 µg/ml); ascorbic acid (0.4 µg/ml) and BDNF (10ng/ml) from day 5 onwards	None
			Neural progenitors	Retinoic acid; Purmorphamine and DAPT	Day 7 onwards for all (10nM; 500nM and 1µM)		
			NMPs	Chiron	Day − 2 to day 7 (2µM OR 4µM OR 6µM)		
Lee et al. [35]	Human (hESCs)	171	Neuroectoderm	bFGF	Day 3 to 7 (20ng/ml)	N2 supplement (1%); B27 supplement (2%); with retinoic acid added from day 7 to 15 (0.1µM). For maturation (from day 15), the concentration of N2 supplement changed to 0.5%	None
			Neural progenitors				
			NMPs	Chiron and SB431542	Day 0 to 3 (2–4µM depending on cell line) and day 0 to 3 (10µM)		
			Interneurons				
			MNs	Purmorphamine	Day 15 to day 21 (1µM)		
			Glial cells				
Zou et al. [40]	Human (hiPSCs)	63	Neuroectoderm	LDN193189; SB431542; FGF2; FGF8 and DAPT	Day 0 to 11 for all (2 µM; 2 µM; 100 ng/ml; 100 ng/ml and 10 µM)	N2 supplement (0.5%); B27 supplement (1%) and ascorbic acid (100mM)	None
			NMPs	Chiron	Day 0 to 8 (3µM)		
			Neural progenitors				
			pMNs and MNs	SAG and Chiron	Day 14 to 22 for both (500nM and 3µM)		
			Mature spinal cord neurons	Retinoic acid; BDNF and GDNF	Day 22 to 25 (1µM); day 22 onwards (10ng/ml) and day 22 onwards (10ng/ml)		
Xue et al. [39]	Human (hESCs and hiPSCs)	56	Neuroectoderm	LDN193189; SB431542 and bFGF	Day 0 to 7 for all (100nM; 10µM and 20 ng/mL)	B27 supplement (2%) and retinoic acid (100nM) added from day 8 to 10. B27 supplement (2%); GDNF (20ng/ml); BDNF (20ng/ml) and Vitamin C (200µM) added from day 21	Porous chitosan microspheres (PCSM) in complex with Matrigel with 0.5mM SAG PCSM-Matrigel@SAG) for sustained release of SAG. 100% Matrigel from day 7
			Spinal cord (caudal) identity	Chiron	Day 0 to 7 (3µM)		
			pMNs and MNs	SAG	Released from matrix		
			Ventral interneurons				
			Dorsal progenitors	BMP4	15ng/ml from day 11 to 20		
			Glial cells				
Chooi et al. [44]	Human (hiPSCs)	120	Neuroectoderm	LDN193189 and SB431542	Day 0 to 10 for both (0.5µM; concentration of SB431542 not stated)	NeuroBrew-21 (1X); N2 supplement (1X). From day 17 onwards, BDNF; GDNF and ascorbic acid were added	Alginate (1% and 2%) from day 10
			Caudal identity	Chiron	Day 0 to 10 (4.25µM)		
			pMNs and MNs	Retinoic acid and Purmorphamine	Day 3 to 10 (10µM) then day 10 to 17 (1µM) and day 10 to 17 (1µM)		
			Ventral interneurons				
			Glial cells				
Balashova et al. [43]	Human (hiPSCs)	21	Neuroectoderm	bFGF	Day 0 to 5 (20ng/ml)	N2 supplement (1%) and heparin (1 µg/ml) from day 5 until day 9. N2 supplement (2%) and B27 supplement without vitamin A (2%); with B27 supplement switched to one containing vitamin A after 4 days	Matrigel (100%) from day 5

pMNs = progenitor motor neurons; MNs = motor neurons; SHH = sonic hedgehog; SAG = smoothened receptor agonist; PEG = polyethylene glycol; NMPs = neuromesodermal progenitors; NCCs = neural crest cells; FGF = fibroblast growth factor; BMP4 = bone morphogenic protein 4; DAPT = N-[N-(3,5-difluorophenacetyl)-l-alanyl]-S-phenylglycine t-butyl ester; BDNF = brain derived neurotrophic factor; GDNF = glial cell line derived neurotrophic factor; CNTF = ciliary neurotrophic factor; IGF-1 = insulin-like growth factor 1; cAMP = cyclic adenosine monophosphate; HGF = hepatocyte growth factor

Despite these limitations, the ability of hNTOs to model human neurodevelopmental events in a sequence analogous to in vivo NT development adds to our understanding of human CNS development and means that they are an ideal high throughput model for the study both of NTDs and other neurodevelopmental defects as well as for drug screening and development. Overall, hNTOs are an exciting prospect that have the ability to overcome the limitations of animal models of neuronal development and can recapitulate species specific developmental timing events in vitro. With refinement and standardization, they have the potential to become the definitive system within which to study neural tube development and defects.

## Data Availability

Not applicable.
